# Status of organophosphate and carbamate resistance in *Anopheles gambiae* sensu lato from the south and north Benin, West Africa

**DOI:** 10.1186/1756-3305-6-274

**Published:** 2013-09-21

**Authors:** Nazaire Aïzoun, Rock Aïkpon, Virgile Gnanguenon, Olivier Oussou, Fiacre Agossa, Gil Germain Padonou, Martin Akogbéto

**Affiliations:** 1 Centre de Recherche Entomologique de Cotonou (CREC), 06 BP 2604, Cotonou, Bénin; 2 Faculté des Sciences et Techniques, Université d’Abomey Calavi, Calavi, Bénin

**Keywords:** *Anopheles gambiae*, *Ace-1*, Resistance, Fenitrothion, Bendiocarb, IRS, Benin

## Abstract

**Background:**

With the rapid spread of pyrethroid resistance in the main malaria vectors from Benin and the various resistance mechanisms involved (metabolic resistance and knock-down resistance (*kdr*), it is important to foresee effective resistance management strategies. Thus, the knowledge of the insensitive acetylcholinesterase (*ace-1*R) effects on phenotypes of *An. gambiae* will help us to strengthen basic and operational research on thedevelopment of strategies that will use organophosphates or carbamates as alternatives against pyrethroids-resistant malaria vectors in the field.

**Methods:**

Larvae and pupae of *Anopheles gambiae s.l.* mosquitoes were collected from the breeding sites in Ouemé, Atacora, and Alibori departments. CDC susceptibility tests were conducted on unfed female mosquitoes aged 2–5 days old. CDC bioassays were performed with stock solutions of fenitrothion (50 μg per bottle) and bendiocarb (12.5 μg per bottle). PCR techniques were used to detect species and *Ace-1* mutations.

**Results:**

* Anopheles gambiae* Seme and Kandi populations were susceptible to fenitrothion whereas *Anopheles gambiae* Tanguieta and Malanville populations were resistant. *An. gambiae* populations from Seme, Kandi and Malanville were fully susceptible to bendiocarb whereas those from Tanguieta have developed a strong resistance to the same insecticide. A slight decrease in mortality rate was observed with 97.91% in populations of mosquitoes from Malanville. PCR revealed that all specimens tested were *Anopheles gambiae s.s..*

The presence of *Ace-1R* at very low frequency (0.01) was observed in *Anopheles gambiae* Malanville populations.

**Conclusion:**

This study demonstrated the need to monitor organophosphate (OPs) and Carbamates resistance among populations of the *An. gambiae s.l.* in Benin, to determine its spread and anticipate vector control failure where these insecticides are used. However, further studies are needed to understand the current distribution of the *Ace-1R* mutation in other localities in the south-north transect Benin.

## Background

Malaria control in Africa is mainly based on the use of indoor residual spraying (IRS) and insecticide-treated nets (ITN) with pyrethroid insecticides essentially because of their knockdown effect, their excito-repellent properties and their low mammalian toxicity [1].

Pyrethroids are the only insecticides currently available for use on bednets. It is clear that, because resistance to these compounds is widespread in Africa [2-5], interest in using IRS (Indoor Residual Spraying) to control malaria vectors is resurging. This IRS strategy is preferentially based on the use of organophosphates and carbamates, either alone or in combination with pyrethroid impregnated bednets [6].

These insecticides inactivate acetylcholinesterase (AChE), an enzyme responsible for neurotransmitter degradation at the cholinergic nerve synapse. However, resistance to OPs and Carbamates based on reduced sensitivity of AChE1 has been detected among *An. gambiae* from Cote d’Ivoire [7,8]. It was also shown that the AChE1 insensitivity reported in *An. gambiae*, *Anopheles albimanus* Wiedemann, and *Culex pipiens* L. was due to the same glycine-serine substitution at position 119 resulting from a single point mutation GGC to AGC in the *ace-1* gene [8,9]. Although the mutation *ace-1* G119S provided cross-resistance to organophosphates and carbamates, the resistance level greatly varied between both insecticide families [10].

The Benin National Malaria Control Programme has implemented indoor residual spraying (IRS) campaign under the financial support of the PMI (President’s Malaria Initiative) using bendiocarb in the north of the country since 2011. The same product was previously used to control *Anopheles gambiae s.l.* populations from Ouemé department in southern Benin (2008-2010).

The present study propose was to assess the resistance status of malaria vectors from Malanville, Kandi, Tanguieta and Seme to bendiocarb and fenitrothion.

Moreover, this study evaluated the presence and extent of the distribution of the *ace-1R* mutation within and among these *An. gambiae s.l.* populations in the south and north Benin, where pyrethroid resistance was also reported in *An. gambiae*[11,12].

## Methods

### Study area

**Figure 1:**
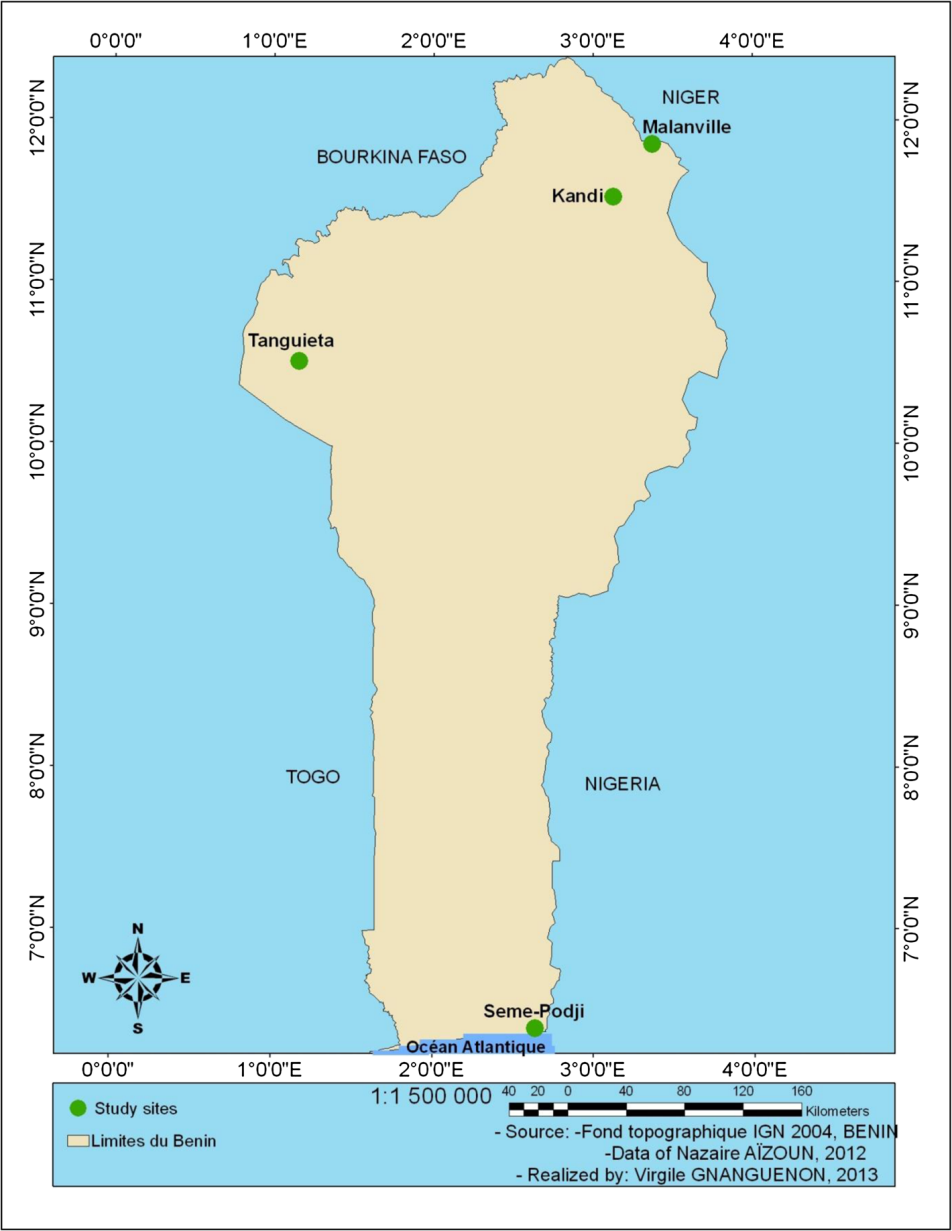
Map of the study area.

The study area is located in Republic of Benin (West Africa) and includes three departments, Ouemé, Atacora and Alibori departments (Figure 1). In Ouemé department located in the South-Eastern Benin, the study was carried out in Seme district recently under IRS with bendiocarb. In Atacora department located in the North-Western Benin, the study was carried out in Tanguieta district under IRS with bendiocarb whereas in Alibori department located in the far north of Benin, the study was carried out in Kandi district, a cotton growing area and in Malanville district, a rice growing area located near the Niger River. The choice of the study sites took into account the economic activities of populations, their usual protection practices against mosquito bites, the indoor residual spraying (IRS) in progress in some of these localities and peasant practices to control farming pests. These factors have a direct impact on the development of insecticide resistance in the local mosquito vectors. Seme is in Ouemé region characterized by a sub-equatorial type of climate with four seasons, two rainy seasons (March-July and September-November) and two dry seasons (December-March and August-September). The temperature ranges from 25 to 30°C with the annual mean rainfall between 900 and 1,500 mm. The northern zone (Tanguieta, Kandi and Malanville) is characterized by a Sudanian climate with only one rainy season per year (May to October) and one dry season (November-April). The temperature ranged from 22 to 33°C with the annual mean rainfall of 1,300 mm.

### Field mosquito collection

*Anopheles gambiae s.l.* mosquitoes were collected during the rainy seasons (March-July and September-November 2012) across Seme district selected in southern Benin. *Anopheles gambiae s.l.* mosquitoes were also collected during the rainy season (May to October) across Tanguieta, Kandi and Malanville districts selected in northern Benin. Larvae and pupae were collected from breeding sites and kept in separated labeled bottles related to each locality. The samples were reared to adults in the CREC (Centre de Recherche Entomologique de Cotonou, Benin) insectary. *Anopheles gambiae* Kisumu, a reference susceptible strain, was used as a control for the bioassay tests. Susceptibility tests were done following a CDC protocol on unfed female mosquitoes aged 2–5 days old, reared from the larval and pupal collections. All susceptibility tests were conducted in the CREC laboratory at 25+/−2°C and 70 to 80% relative humidity.

### Testing insecticide susceptibility

Females *An. gambiae* aged 2 to 5 days old were exposed to the CDC diagnostic dosage of various insecticides according to the CDC protocol [13]. The following insecticides were tested: fenitrothion (50 μg per bottle) and bendiocarb (12.5 μg per bottle). Mosquitoes were exposed for two hours to insecticide-treated bottles and monitored at different time intervals (15, 30, 35, 40, 45, 60, 75, 90, 105, 120 minutes). This allowed us to determine the percentage of total mortality (Y axis) against the exposure time (X axis) for all replicates using a linear scale. Dead and surviving mosquitoes were separately stored in individual tubes with silicagel and preserved at -20°C in the laboratory, for further molecular characterization.

### PCR detection of species and *Ace-1* mutations

Specimens of *An. gambiae* from the CDC bioassay tests were subjected to the *An. gambiae* species specific PCR assays for species identification [14]. The PCR-RFLP diagnostic test was used to detect the presence of G119S mutation (*ace.1R* gene) as described by Weill *et al.*[8]. Mosquito genomic DNA was amplified using the primers Ex3AGdir 5′GATCGTGGACACCGTGTTCG3′ and Ex3AGrev 5′AGGATGGCCCGCTGGAACAG3′ according to Weill *et al.*[8]. One microlitre of total DNA extracted from a single mosquito was used as a template in a 25 μl PCR reaction containing Taq DNA polymerase buffer, 0.2 mM dNTP and 10 pmol of each primer. The PCR conditions were 94°C for 5 min and then 35 cycles of (94°C for 30 s, 54°C for 30 s and 72°C for 30 s) with a final 5 min extension at 72°C. Fifteen microlitres of PCR product were digested with 5U of AluI restriction enzyme (Promega) in a final volume of 25 μl. The PCR fragments were fractionated on a 2% agarose gel stained with ethidium bromide and visualized under UV light.

### Statistical analysis and data interpretation

The resistance status of mosquito samples was determined according to the CDC Criteria [13,15]. The susceptibility thresholds at the diagnostic time of 30 minutes for both organophosphates and carbamates are:

Mortality rate = 100%: the population is fully susceptible

Mortality rate < 100%: the population is considered resistant to the tested insecticides.

Abbott’s formula was not used in this study for the correction of mortality rates in test-bottles because the mortality rates in all controls was always less than 5% [16].

To compare the status of insecticide resistance, Fisher’s exact test was carried out to determine if there was any significant difference between mortality rates of populations of *An. gambiae s.s.* of districts using Statistica 6.0. Allelic frequencies of G119S mutation were analysed using the version 1.2 of Genepop [17]. To assess if the mutation frequencies were identical across populations, the test of genotypic differentiation was performed [18].

### Ethical approval

This study was approved by the Ministry of Health and the Center for Entomological Research of Cotonou.

## Results

### Susceptibility of *An. gambiae s.l.* populations to fenitrothion and bendiocarb

Kisumu strain (control) confirmed its susceptibility status with 100% mortality as a reference strain. All female mosquitoes of *Anopheles gambiae* Kisumu, which were exposed to CDC bottles treated with fenitrothion 50 μg/bottle and bendiocarb 12.5 μg/bottle showed a total mortality with no survivors after 30 minutes of exposure. This result represents a susceptibility threshold time or diagnostic time as defined by CDC protocol (Table 1).

**Table 1:** **Mortality of *Anopheles gambiae *from the districts of Malanville, Kandi Tanguieta, and Seme-Kpodji after 2 hours exposure to fenitrothion (50 μg/bottle) and bendiocarb (12.5 μg/bottle)**

**Localities**	**Insecticides**	**Number tested**	**% Mortality**	**Resistance status**
Kisumu (Control)	Fenitrothion	33	100	S
	Bendiocarb	26	100	S
Malanville	Fenitrothion	14	57.14	R
	Bendiocarb	48	97.91	S
Kandi	Fenitrothion	50	100	S
	Bendiocarb	42	100	S
Tanguieta	Fenitrothion	116	76.72	R
	Bendiocarb	76	78.94	R
Seme	Fenitrothion	78	100	S
	Bendiocarb	85	100	S

A percentage mortality of 57.14% of *An. gambiae* from Malanville and 76.72% of *An. gambiae* from Tanguieta were recorded after 30 minutes of exposure to CDC bottles treated with fenitrothion. These mortalities below the CDC protocol threshold indicate that populations of *An. gambiae* from Malanville and Tanguieta were resistant to fenitrothion. Following exposure of *An. gambiae* from Kandi and Seme populations to CDC fenitrothion treated bottles, 100% mortality was recorded on both strains suggesting a complete susceptibility of these populations to the OP fenitrothion (Table 1).

Regarding *An. gambiae* from Tanguieta site, a mortality of 78.94% was recorded after exposure to CDC bottles treated with bendiocarb showing an indication of resistance of this population of mosquitoes to bendiocarb. On the one hand *An. gambiae* from Malanville showed 97.91% mortality after 30 minutes of exposure to bendiocarb which is suggestive of its resistance to this product whereas *An. gambiae* from both Kandi and Seme on the other hand gave 100% mortality indicating a total susceptibility to this carbamate (Table 1).

### Species of *Anopheles gambiae* and *Ace-1* genotype

PCR revealed 100% of mosquitoes tested were *Anopheles gambiae s.s.* The frequencies of *Ace-1R* in *Anopheles gambiae* Kandi and Malanville were 0% and 1% respectively (Table 2).

**Table 2:** ***Ace-1 *mutation frequency in *An. gambiae *populations issue from performing CDC bioassays tests**

**Localities**	**Number tested**	**Species Ag**	***Ace-1 *mutation**			
			**RR**	**RS**	**SS**	**F( *Ace-1 *)**
Kandi	48	48	0	0	48	0
Malanville	48	48	0	1	47	0.01

Ag An. gambiae s.s.

## Discussion

Pyrethroid-treated bednets remain one of the major tools for malaria vector control in tropical areas. However, the spectrum of resistance to pyrethroids calls for alternative and complementary solutions. Even if they are not recommended for bednets impregnation, OPs and Carbamates should be considered as alternatives for indoor residual sprayings.

The *Ace-1R* frequency observed in *Anopheles gambiae* Malanville populations (0.01) showed the need to monitor OPs and Carbamates resistance among these *An. gambiae* populations which were resistant to fenitrothion. The slight decrease of susceptibility obtained with *Anopheles gambiae* Malanville populations exposed to bendiocarb was not synonymous with resistance. A similar pattern was already observed with *Anopheles gambiae* Adjara and Dangbo populations exposed to bendiocarb in Ouemé department in southern Benin [19]. Our study confirmed the recent study conducted by Djègbé *et al.*[12] who showed that *Anopheles gambiae* Malanville is still susceptible to bendiocarb. However, the current study may contradict the study of Corbel *et al.*[20] who showed that *Anopheles gambiae* Malanville were resistant to carbosulfan, a carbamate, with the mortality rate of 75%. In addition, in the current study, PCR revealed that 100% of mosquitoes from Malanville tested were *Anopheles gambiae s.s.* Conversely, Corbel *et al.*[20] have shown that *Anopheles arabiensis* populations were also present in Malanville.

In the current study, *Anopheles gambiae* Kandi populations were susceptible to bendiocarb whereas Djogbenou *et al.* [2008] (and unpublished data) have shown that *Anopheles gambiae* Kandi populations were resistant to carbosulfan, a carbamate, with a mortality rate of 73%. There are no previous published studies about the resistance status of *An. gambiae* populations from Kandi district to carbamates and OPs until a recent report by Aïkpon *et al.* 2013. Therefore, these populations of *An. gambiae* need to be monitored for insecticide resistance in this area.

Two years after the fourth round (last round) of IRS with bendiocarb in Ouemé department, *Anopheles gambiae* Seme populations remained susceptible to bendiocarb and fenitrothion. Our study confirmed the recent study conducted by Padonou *et al*[21] who have demonstrated that *Anopheles gambiae* Seme populations were still highly susceptible to bendiocarb with a mortality rate more than 99%. The *Ace-1R* frequency found by these authors was 0.13 in M form of *Anopheles gambiae s.s.* from Seme district. However, this *Ace-1R* frequency needs to be monitored.

*Anopheles gambiae* Tanguieta populations were resistant to fenitrothion and bendiocarb**.** Previous field survey in *An. gambiae s.l.* populations of South-Western Burkina Faso, where a high amount of insecticides was used in cotton growing areas, showed that *ace-1R* mutation was the main resistance mechanism of *An. gambiae s.l.* to carbamates and organophosphates [22].

Although this mechanism was not investigated in *Anopheles gambiae* Tanguieta populations in the current study, a recent study showed that the *ace-1R* mutation was present in *Anopheles gambiae* Tanguieta populations resistant to bendiocarb in North-Western Benin [23]. These authors have reported throughout their study that there was a cross-resistance between OPs (fenitrothion) and carbamates (bendiocarb and propoxur) in *Anopheles gambiae* Tanguieta populations with the presence of *Ace-1R* at low frequency: 0.12 in S molecular form and 0.08 in M molecular form. Thus, our study confirms the study of Aïkpon *et al*[23]. In addition, esterases might also play a little role in *Anopheles gambiae* Tanguieta populations resistant to bendiocarb [24].

Once again, complementary studies are needed to precisely determine which resistance mechanism leads to the decrease of mortality against bendiocarb, its current frequency and geographic distribution in field populations of *An. gambiae s.l.* of Benin. This is particularly relevant for campaigns of indoor residuals spraying based on carbamate and/or organophosphate that are currently implemented by the National Malaria Control Programme in the northern part of the country after the Ouemé campaigns in South-Eastern Benin ended in 2010. Currently, the IRS strategy in progress in Atacora department is preferentially based on the use of organophosphates and carbamates and the spread of the *ace-1R* mutation could represent a major threat to the effectiveness of this strategy. *Ace-1R* mutation occured in *Anopheles gambiae* Tanguieta populations in Atacora department located in North-Western Benin was already found in many specimens of *An. gambiae s.s.* in South-Western Burkina Faso [22] and in addition to the case of Bouake in Côte d’Ivoire [7]. All these findings suggest that OP and Carbamate resistance may be widespread throughout West Africa.

This wide distribution of *ace-1R* mutation in West Africa theoretically could result either from the spread of the single mutation or from the independent occurrence of the same or different mutations in many countries. Although the rate of mutations generating resistance genes among mosquitoes is unknown, we can consider that the migration including passive transportation of mosquitoes and gene flow play major roles in the dispersion of resistance genes between distant populations [25]. It is clear that closer collaboration between resistance experts in agriculture and public health is needed. Public health agencies can definitely benefit from the extensive experience gained by the agricultural sector in promoting integrated pest-management principles as well as disseminating simple and pragmatic guidelines for insecticide resistance management.

## Conclusion

The resistant allele *ace-1R* confers resistance to organophosphates and carbamates compounds. This resistance potentially represents a threat to the implementation of malaria prevention programmes based on the use of insecticides. Given the importance of the vector control against malaria disease, there is an urgent need for field and laboratory monitoring of insecticide resistance. Characterization of the biochemical interactions between insecticides and resistant target sites will contribute to identifying or to designing new insecticides that should improve effectiveness of resistance management strategies against resistant *Anopheles* species in tropical regions. Further genetic studies need to be performed on mosquito samples to investigate *Ace-1 R* gene flow throughout West Africa countries including Benin country. Understanding or predicting the spread of insecticide resistance genes into mosquito populations of the *An. gambiae* complex will be crucial for the development of effective methods to control the main malaria vector in Africa.

## Competing interests

The authors declare that they have no competing interests.

## Authors’ contributions

MA and NA conceived the study. GGP and RA participated in the design of the study. Entomologic data was collected by NA, VG, OO, FA and bioassays and laboratory analysis were carried out by NA, VG and OO. MA and NA participated in the analysis and interpretation of data. VG has contributed to the mapping. The manuscript was drafted by NA and MA has been involved in revision of the manuscript. All authors read and approved the final manuscript.
